# Arabidopsis Response to Inhibitor of Cytokinin Degradation INCYDE: Modulations of Cytokinin Signaling and Plant Proteome

**DOI:** 10.3390/plants9111563

**Published:** 2020-11-13

**Authors:** Veronika Berková, Michaela Kameniarová, Vladěna Ondrisková, Miroslav Berka, Simona Menšíková, Romana Kopecká, Markéta Luklová, Jan Novák, Lukáš Spíchal, Aaron M. Rashotte, Břetislav Brzobohatý, Martin Černý

**Affiliations:** 1 Department of Molecular Biology and Radiobiology, Faculty of AgriSciences, Mendel University in Brno, 61300 Brno, Czech Republic; veronikamalych@gmail.com (V.B.); mikameni@gmail.com (M.K.); ondriskova@vupt.cz (V.O.); miroslavberka94@gmail.com (M.B.); simensikova@gmail.com (S.M.); romcako@gmail.com (R.K.); luklovam@gmail.com (M.L.); novakhonza@atlas.cz (J.N.); brzoboha@ibp.cz (B.B.); 2 Department of Chemical Biology and Genetics, Centre of the Region Haná for Biotechnological and Agricultural Research, Faculty of Science, Palacký University, 77200 Olomouc, Czech Republic; lukas.spichal@upol.cz; 3 Department of Biological Sciences, Auburn University, Auburn, AL 811, USA; rashotte@auburn.edu; 4 Central European Institute of Technology, Faculty of AgriSciences, Mendel University in Brno, 61300 Brno, Czech Republic

**Keywords:** cytokinin, CKX, inhibitor of cytokinin degradation, proteome, Arabidopsis thaliana, stress response attenuation

## Abstract

Cytokinins are multifaceted plant hormones that play crucial roles in plant interactions with the environment. Modulations in cytokinin metabolism and signaling have been successfully used for elevating plant tolerance to biotic and abiotic stressors. Here, we analyzed *Arabidopsis thaliana* response to INhibitor of CYtokinin DEgradation (INCYDE), a potent inhibitor of cytokinin dehydrogenase. We found that at low nanomolar concentration, the effect of INCYCE on seedling growth and development was not significantly different from that of trans-Zeatin treatment. However, an alteration in the spatial distribution of cytokinin signaling was found at low micromolar concentrations, and proteomics analysis revealed a significant impact on the molecular level. An in-depth proteome analysis of an early (24 h) response and a dose-dependent response after 168 h highlighted the effects on primary and secondary metabolism, including alterations in ribosomal subunits, RNA metabolism, modulations of proteins associated with chromatin, and the flavonoid and phenylpropanoid biosynthetic pathway. The observed attenuation in stress-response mechanisms, including abscisic acid signaling and the metabolism of jasmonates, could explain previously reported positive effects of INCYDE under mild stress conditions.

## 1. Introduction

Plants are sessile organisms that, in order to maintain growth and survival under harsh conditions, have evolved unique mechanisms, enabling them to react rapidly to ever-changing ambient conditions. A fundamental part of this mechanism is the plant hormone cytokinin, which is known to function in many different abiotic and biotic stress responses [1,2,3]. While cytokinin homeostasis is necessary for optimal growth and development, it has been demonstrated that an increase in the cytokinin pool may serve to improve crop yield [4,5,6]. A short period of stress has been shown to elevate levels of active cytokinins [7]. However, this effect is only transient, and extended stress conditions usually result in a significant reduction of the active cytokinin pool [8,9]. 

Plant cytokinin levels can be modulated by the application of exogenous hormones, and several studies have found a positive effect of cytokinin treatment on crop production (e.g., [10,11]). However, the intricacy of cytokinin’s effects has limited the use of such a technique in agriculture to date [12]. Endogenous cytokinin content is known to be regulated by both novel biosynthesis and reversible and irreversible conjugation into compounds with different levels of activity and distribution within the plant, and by the ability to be degraded into inactive products [13,14]. Cytokinin dehydrogenase (CKX) is the main enzyme that catalyzes the inactivation by irreversible degradation of cytokinins. In Arabidopsis, CKX is encoded by seven genes with different substrate specificity, spatial and temporal expressions, and subcellular targeting into the cytosol (CKX7), vacuole (CKX1, CKX3) and endoplasmic reticulum or apoplast (CKX2, CKX4-6) [15,16,17,18]. Manipulating CKX activity presents a potentially useful tool for enhancing crop resistance against adverse environmental conditions and improving plant production. The overexpression of CKX genes leads to cytokinin-deficiency phenotype, such as an enlarged root system, reduced activity of the vegetative and floral shoot apical meristem, and an earlier differentiation of plastids and reduction of chlorophyll content in leaves [15,19]. On the other hand, CKX-deficient mutants showed an increased number of reproductive organs and higher seed yield in Arabidopsis, rice, barley or wheat [5,6,20,21].

Pharmacological treatment is a suitable and GMO-free approach for inhibiting CKX activity; INCYDE [2-chloro-6-(3-methoxyphenyl)aminopurine] is one of the compounds with a high affinity for the CKX enzyme and only a low-level activation of AHK cytokinin receptors [22]. The INCYDE inhibition of CKX has been successfully demonstrated, both under controlled conditions and in field studies, and it reportedly improved plant resistance to diverse biotic and abiotic stresses, including salinity, heat stress recovery, heavy metal toxicity and *Verticillium longisporum* infection [23,24,25,26]. It is believed that this positive effect on plant resilience is predominantly the result of cytokinin accumulation, but the exact molecular mechanisms are far from being understood. In this study, the impact of INCYDE treatment on the model plant *Arabidopsis thaliana* was analyzed. We compared its effect to that of a major active cytokinin base trans-Zeatin (tZ) and provide insights into the proteome response to short- and long-term INCYDE exposure.

## 2. Results

### 2.1. Root Growth in Response to tZ and INCYDE Treatment was not Significantly Different

**Figure 1:**
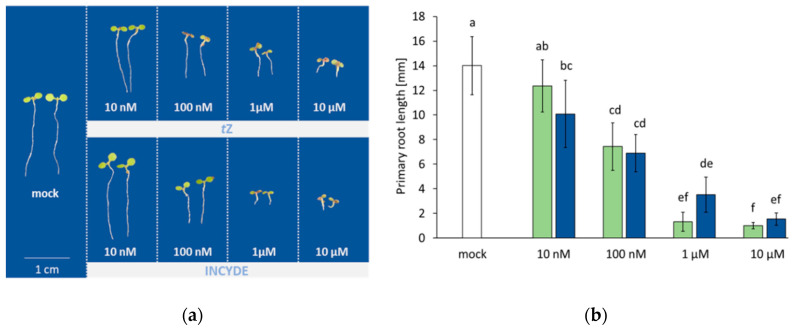
INCYDE and tZ showed a similar effect on seedling development. (**a**) Images of two representative seven-day-old seedlings and (**b**) the comparison of root elongation in the presence of INCYDE (green), tZ (blue) or mock (white). Results represent means and standard deviation (*n* = 30), different letters indicate significant differences (Kruskal-Wallis, *p* < 0.05).

First, to compare the effects of exogenously applied tZ and INCYDE on Arabidopsis physiology, we monitored the primary root growth. Root growth inhibition is a well-known response to cytokinin (e.g., [27]). Seedlings were cultivated as described in Materials and Methods, and after seven days, the length of primary roots was evaluated (Figure 1a). The inhibition of root growth was already observed at the lowest applied concentration (10 nM), and both substances fully retarded seedling growth at 10 µM, which was also accompanied by cotyledon coloring due to the accumulation of anthocyanin (a cytokinin response described in previous studies, e.g., [28]). The INCYDE treatment had a slightly lower effect at 10 nM, but the saturating concentration was lower than that of tZ (Figure 1b).

### 2.2. INCYDE Treatment Elicited Distinct Spatial Distribution of Cytokinin Signaling

**Figure 2:**
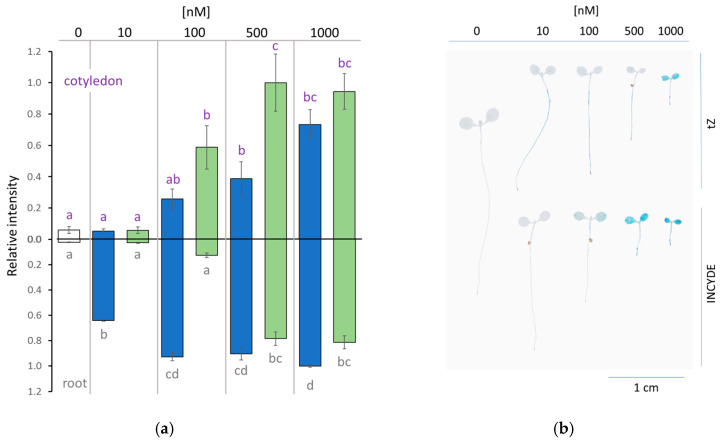
INCYDE induced strong cytokinin signaling response in cotyledons. (**a**) The comparison of normalized ARR5 promoter activity visualized by histochemical staining and (**b**) representative images of seven-day-old ARR5::GUS reporter line cultivated on the medium supplemented with trans-Zeatin (tZ, blue; *n* = 16), INCYDE (green; n=20) or DMSO (mock, white; *n* = 16). Results represent means and standard error, different letters indicate significant differences (Kruskal-Wallis, *p* < 0.05; see Table S1 for details).

Next, the effect of INCYDE and exogenously supplied tZ on cytokinin signaling was compared. Transgenic Arabidopsis lines bearing ARR5::GUS reporter were grown within the concentration range of INCYDE, and after seven days, the ARR5 promoter activity was visualized by histochemical staining and compared to that of tZ (Figure 2a,b). The observed differences between tZ and INCYDE were concentration-dependent. The analysis revealed that the growth of seedlings in the presence of INCYDE led to a higher increase in the ARR5 promoter activity in cotyledons (100–500 nM) but a lower cytokinin signaling in the roots (10–100 nM). The most promising response was found for seedlings grown in the presence of 500 nM tZ and INCYDE, with a similar increase in the ARR5 promoter activity in the roots, but a strikingly different pattern in cotyledons (Figure 2). A similar effect was observed in 14-day-old plants (Figure S1).

### 2.3. Early INCYDE Response Proteins of Arabidopsis Seedlings Highlight Similarity to Exogenous tZ Treatment 

**Figure 3:**
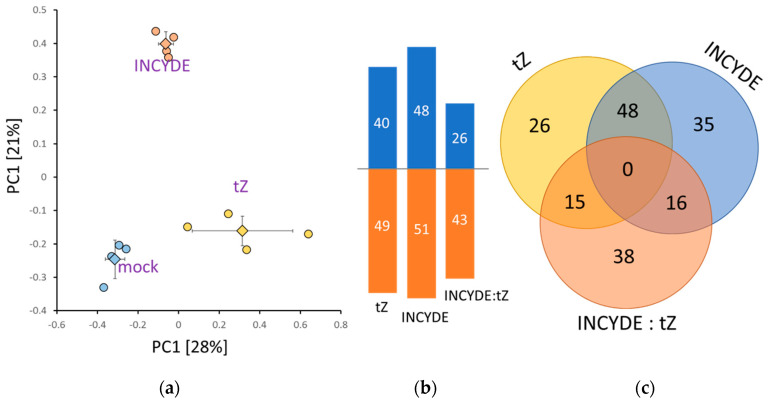
Early INCYDE- and tZ-responsive proteins in Arabidopsis. (**a**) The proteome profile separation after 24 h incubation with 500 nM INCYDE or tZ. Principal component analysis based on quantitative data of 178 differentially abundant proteins. Results of four biological replicates, including means and standard deviation; (**b**) Differentially abundant proteins accumulated (blue) and decreased (orange) compared to mock (tZ—tZ vs. mock; INCYDE—INCYDE vs. mock) or tZ-treated samples (INCYDE:tZ); (**c**) Overlap between tZ and INCYDE-responsive proteins. For details, see Supplementary Table S2.

The dose-dependent growth response to INCYDE indicated that the most interesting comparison between tZ and INCYDE treatment was within the 100–1000 nM range (Figure 1). To provide an insight into the molecular mechanisms behind the observed contrasting response, seven-day-old seedlings were treated for 24 h with 500 nM tZ, INCYDE, or 0.1% (*v/v*) dimethylsulphoxide (DMSO, mock), as described in Materials and Methods, to determine an early response. In total, 3273 Arabidopsis proteins were identified with reliable quantitative data for more than 2100 of these. Higher biological variability for tZ was observed within the set of four biological replicates (each pooled from at least 20 seedlings), but statistically significant (*p* < 0.05) separation between tZ, INCYDE and mock-treated samples was apparent (Figure 3a). A detailed analysis revealed 89 and 99 tZ and INCYDE early-response proteins as compared to mock-treatment, respectively, and 69 proteins that showed statistically significant and reproducible differences between INCYDE- and tZ-treated samples (Figure 3b).

**Figure 4:**
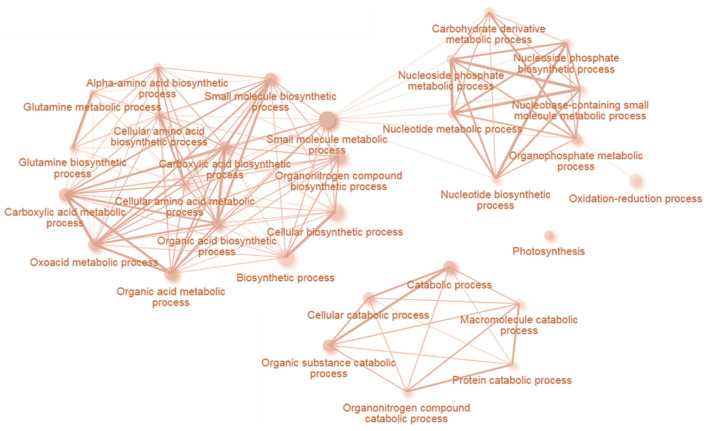
Visualization of processes mediated by early INCYDE response proteins. Enrichment analysis based on hypergeometric distribution followed by FDR correction. Two pathways are connected if they share 20% or more proteins. Darker and bigger nodes are more significantly enriched and larger sets, respectively. Analyzed by ShinyGO 0.61 [32].

As illustrated in Figure 4, early INCYDE response proteins were functionally enriched in amino acid and carbohydrate metabolism, but the set encompassed diverse processes of both primary and secondary metabolism. The comparison with tZ-responsive proteins showed only 48 shared proteins, but all with a similar responsiveness. Repressed proteins included an enzyme of anthocyanin metabolism leucoanthocyanidin dioxygenase (AT4G22880), senescence-associated protein SAG24 (AT1G66580) and a protein reportedly involved in the regulation of gravitropic response and auxin transport in roots ROSY1 (AT2G16005, [29]). Significantly accumulated proteins included those required for chloroplast biogenesis and development (chorismate mutase 3, AT3G29200; CPP1, AT5G23040, [30]), enzymes involved in cell wall formation (AT5G15490, AT4G37800; [31]), chloroplastic and cytosolic isoform of glutamine synthetase (AT5G35630, AT1G66200), and extracellular protein with a putative role in circadian rhythm and abiotic stress GER3 (AT5G20630).

The set of 33 INCYDE-repressed proteins contained eight enzymes involved in secondary metabolism, including phenylalanine ammonia-lyase 1 (AT2G37040), chalcone-flavanone isomerase (AT5G05270) and an enzyme of jasmonic acid biosynthesis (allene oxide cyclase; AT3G25770). INCYDE elicited accumulation of 18 proteins, including a zinc metalloprotease FTSH4 (AT2G26140) involved in assembly and stability of the mitochondrial complex V, a protein of retrograde signaling PRIN2 (AT1G10522), and two proteins associated with intracellular protein trafficking and endocytosis (RABG3c, AT3G16100; AGD8, AT4G17890).

A comparison between INCYDE-responsive and tZ-responsive proteins highlighted an INCYDE-induced elevation of several ribosomal proteins and enzymes of ROS-metabolism (superoxide dismutase AT2G28190; alcohol dehydrogenase AT1G77120; ascorbate peroxidase AT1G07890; glutathione S-transferase AT2G29450; thioredoxin M4, AT3G15360; peroxidase AT4G30170). Changes in ROS-metabolism could imply higher INCYDE toxicity.

### 2.4. Correlation between INCYDE Concentration and Protein Abundance

**Figure 5:**
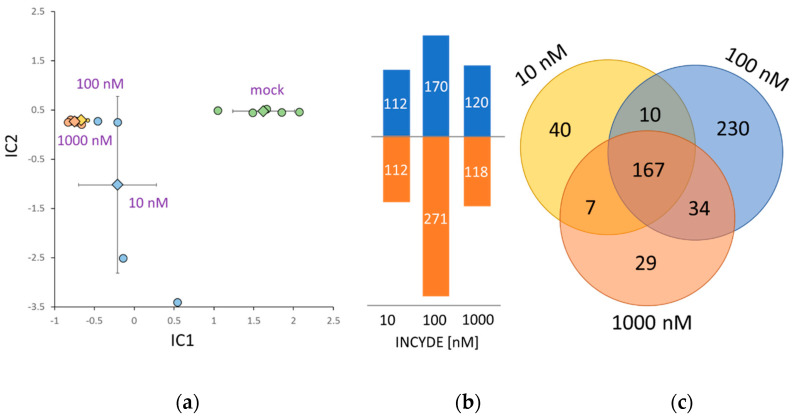
Dose-dependent INCYDE response of Arabidopsis proteome. (**a**) The proteome profile separation of INCYDE- and mock-treated samples. Independent component analysis based on quantitative data of 1500 most abundant proteins. Results of five biological replicates, including means and standard deviation; (**b**) Accumulated (blue) and decreased (orange) INCYDE-responsive proteins; (**c**) Overlap between the differentially-abundant proteins found in response to indicated INCYDE concentrations. For details, see Supplementary Table S3.

Next, an INCYDE dose-response was evaluated at a proteome-wide scale. Seven-day-old seedlings cultivated on textile meshes were transferred onto new medium supplemented with 10-1000 nM INCYDE or 0.1% (*v*/*v*) DMSO (mock), as described in Materials and Methods. After seven days, shoots were collected for proteome and metabolome analyses. The effect of INCYDE on the growth of fully established seedling was significantly lower than that observed at the early developmental stage (Supplementary Figure S2), but its effect was well-manifested at the proteome level. In total, 29,530 peptide groups were identified, providing sufficient quantitative data for more than 2400 proteins. Independent component analysis (Figure 5a) showed significant separation of INCYDE- and mock-treated samples, and indicated a high level of similarity between 100 and 1000 nM INCYDE treatments. A detailed comparison of differentially abundant proteins (absolute fold-change 1.4, *p* < 0.05) confirmed the expected overlap between the INCYDE treatments, and revealed that the highest response was elicited at an INCYDE concentration of 100 nM. The correlation analysis identified 25 and 11 statistically significant positive and negative correlations, respectively (absolute Pearson’s correlation coefficients >0.7, *p* < 0.01). These INCYDE-responsive proteins are involved in signaling, primary and secondary metabolism, including γ-aminobutyric acid biosynthesis, glucosinolate degradation and processes of chloroplast biogenesis and development (Table 1).

### 2.5. INCYDE Response Proteins Modulate Diverse Metabolic Processes

**Figure 6:**
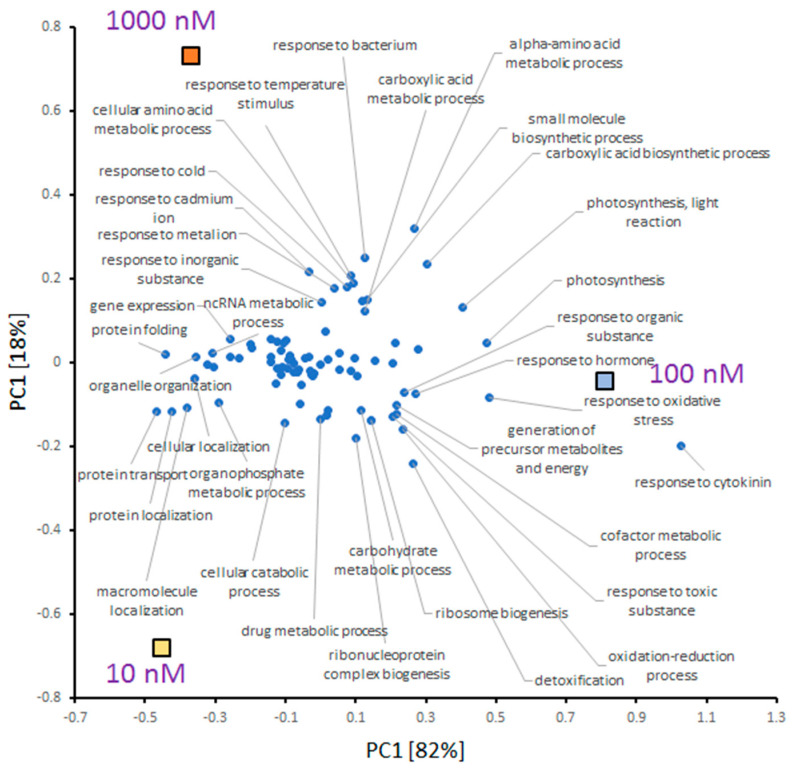
Comparison of enriched biological processes in INCYDE-responsive proteomes. For the sake of clarity, categories with at least 15 proteins were included in the final analysis, and only GO terms with the most significant contribution to the separation in PC1 or PC2 are labeled. The separation of individual treatments (represented by boxes) indicates a similarity between 10 and 1000 nM samples.

In total, 517 proteins representing an estimated 29% of the total protein extract were found to be INCYDE-responsive (Figure 5b,c). A functional analysis revealed 199 enriched biological processes (Gene Ontology; GO), covering 448 INCYDE-responsive proteins. The strongest response was detected for 100 nM INCYDE treatment, and the GO enrichment (Figure 6) revealed that this response elicited the highest similarity to annotated response to cytokinin. Other highly enriched categories compared to 10 and 1000 nM treatments included ‘response to oxidative stress’, ‘photosynthesis’, ‘response to light stimulus’ and ‘ribosome biogenesis’.

There were only 40 INCYDE-responsive proteins found exclusively for the 10 nM treatment. Besides proteins associated with proteosynthesis (7), photosynthesis (5) and ubiquitin-mediated degradation (6), two INCYDE-responsive proteins are reportedly involved in biotic stress response, namely inhibitor of fungal polygalacturonase PGIP1 (AT5G06860; accumlated) and extracellular lectin, whose expression is induced upon treatment with chitin oligomers (AT3G16530; repressed), and at least three repressed proteins participate in intracellular trafficking (AT5G16880, AT5G05010, AT5G16880). The lowest number of dose-specific proteins was found for 1000 nM treatment (Figure 5c). The notable INCYDE-responsive proteins included fungal growth inhibitor PR4 (AT3G04720, repressed), an enzyme of glucosinolate degradation (AT5G26000, positive correlation with INCYDE dosage, Table 1) and two heat shock proteins HSP70 (AT3G12580, repressed; AT5G42020, accumulated).

**Figure 7:**
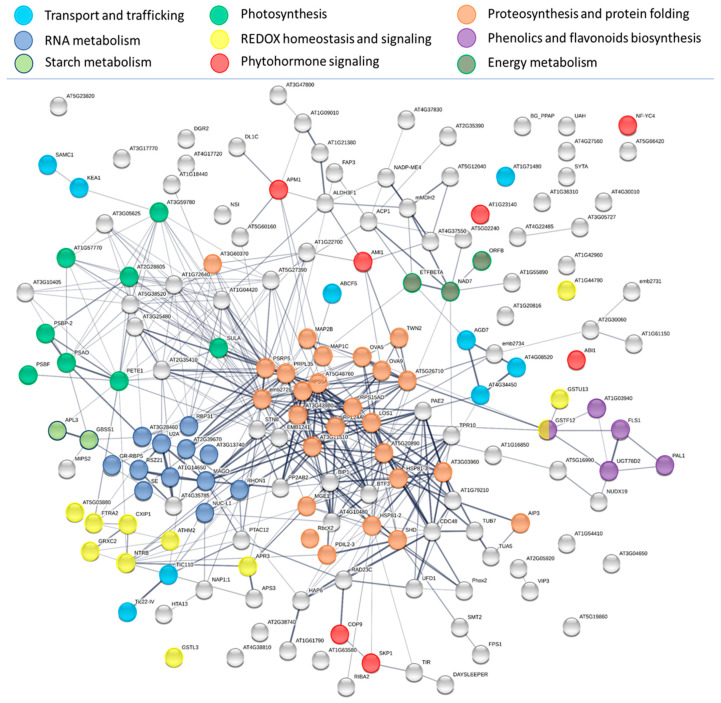
Proteins with the dose-independent response to INCYDE. Interactions and functional clusters of INCYDE-responsive proteins highlighted by String [37]; Color coding of proteins is denoted by functional designation given by GO and KEGG pathway enrichments; only the nine most relevant categories are highlighted. The line thickness indicates the strength of data support, the minimum required interaction score is 0.4 (medium confidence).

Next, the proteins found with a similar INCYDE response in all three treatments were analyzed. The dose-independent changes in the amounts of at least ten proteins involved in RNA metabolism, and the accumulation of three proteins involved in chromatin remodeling (H2A.2, AT3G20670, nucleosome assembly protein AT4G26110, a protein involved in histone modifications VIP3, AT4G29830), clearly demonstrated an impact of INCYDE on expression and transcription machinery. A significant portion of INCYDE-responsive proteins found in all three sets was involved in proteosynthesis, ribosome biogenesis, tRNA metabolism and protein folding (Figure 7). The accumulation of phenylpropanoid and flavonoid biosynthetic enzymes was recently reported in barley root proteome response to tZ [33]. Here, phenylalanine ammonia-lyase 1, flavonol synthase and anthocyanidin 3-O-glucosyltransferase were significantly accumulated in response to INCYDE. Cytokinin signaling is intertwined with other phytohormonal networks, and especially that of auxin. It is thus not surprising that the INCYDE treatment resulted in the accumulation of auxin biosynthetic enzyme (AMI1, AT1G08980) and a putative negative regulator of PIN auxin transport (APM1, AT4G33090). All tested concentrations of INCYDE also elicited a depletion of abscisic acid (ABA) signaling transcription factor NFYC4 (AT5G63470), and accumulations of ABA signaling repressor phosphatase 2C (ABI1, AT4G26080) and protein CAR8 (AT1G23140). CAR8 is a putative mediator of ABA receptor interaction with the plasma membrane and could regulate sensitivity to ABA [34]. Several proteins were also connected to photomorphogenesis and plastid biogenesis, including a subunit of COP9 signalosome complex (AT4G14110, decreased), PTAC12 (AT2G34640, accumulated) which is reportedly involved in the initiation of photomorphogenesis [35], epimerase participating in plastid division AT2G21280 and protein CLMP1 (AT1G62390, accumulated), that is required for plastid separation and partitioning during cell division [36]. Last but not least, farnesyl pyrophosphate synthase 1 (AT5G47770) was significantly repressed, which may coincide with the expected repression in the biosynthesis of isopentenyl pyrophosphate, a substrate of cytokinin biosynthetic enzyme isopentenyl transferase.

## 3. Discussion

### 3.1. Cytokinin Dehydrogenase Isoforms Play a Key Role in the Contrasting Response between tZ and INCYDE

**Figure 8:**
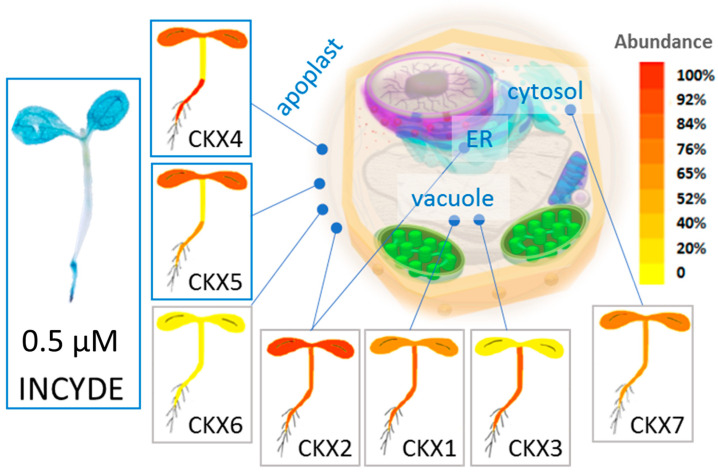
Organ-specific localization of apoplastic CKX isoforms CKX4 and CKX5 correlates with ARR5 promoter activity at 0.5 µM INCYDE. Protein iBAQ data from *Arabidopsis thaliana* expression atlas that correlate with protein abundances were visualized with an electronic fluorescent pictograph (100% corresponds to the iBAQ value 25.0); Expected subcellular localizations are indicated [38,40].

The effect of exogenously applied cytokinin strongly depends on its transport and metabolism. It is thus not surprising that the direct tZ effect on the cytokinin signaling was predominantly constrained to the root tissue (Figure 2). In contrast, the mechanisms of rapid cytokinin inactivation and degradation did not affect INCYDE transport, and the INCYDE induced cytokinin response was determined by the presence of CKX enzymes. Components of cytokinin metabolism and signaling are low abundant proteins, and the quantitative data for CKX were not available in any of the INCYDE response datasets. However, the recently published detailed characterization of Arabidopsis proteome [38] implied that the amounts of cytokinin receptor in root, hypocotyl and cotyledon are comparable, and showed that the observed activation of ARR5::GUS promoter (Figure 2) was similar to the expected profiles of apoplastic isoforms CKX4 and CKX5 (Figure 8). The apoplastic CKX enzymes found in Arabidopsis act mainly on cytokinin free bases and ribosides [16], and these are also the root-to-shoot long-distance signaling forms of cytokinin [39]. It is thus possible that at a given developmental stage, the majority of the INCYDE activity comprises the transport of active cytokinin in plants.

### 3.2. Cytokinin Response Targets Ribosomal Proteins

**Figure 9:**
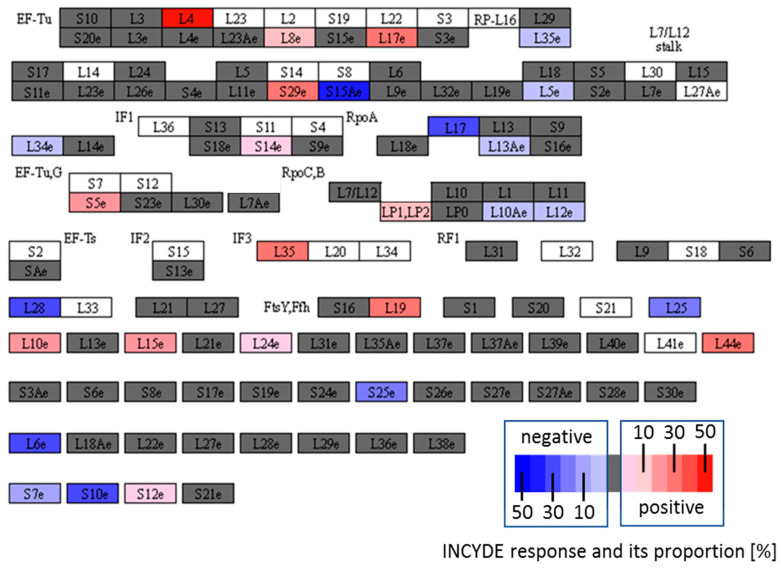
INCYDE response ribosomal proteins. A graphical representation of all identified ribosomal subunits in shoots of plants exposed to INCYDE for 168 h. Annotated using the KEGG Mapper [45]. Blue and red gradients indicate subunits with one or more INCYDE-responsive paralogs. The contribution of the paralog to the subunit composition [%] is indicated; white—not detected; grey—no significant difference compared to the mock-treated plants.

Plant ribosomes are highly complex structures, with each ribosomal protein being encoded by two to seven paralogs. The ribosome composition reflects external stimuli, and may have a significant impact on plant responses [41]. Previous proteomics reports have indicated that ribosome could be the target of cytokinin signaling [33,42,43]. Here, 122 and 167 ribosomal proteins were identified in seedling and shoot proteome, respectively. First, 60S ribosomal proteins L4-1 and L4-2, previously implicated in response to cytokinin [43], were quantified in both experiments, but there was not any detectable difference compared to the mock-treated plants (Figure 9, L4e). The abundances of only three ribosomal proteins were significantly altered in early INCYDE response. Interestingly, a decrease in protein abundance was observed for 60S ribosomal protein L10-3 involved in UV-B stress response [44], and a previous analysis found expression profile similarities between UV-B receptor and components of cytokinin signaling [2]. The experiment with the dose-dependent response to INCYDE revealed a much higher response in ribosomal proteins. In total, 16 and 18 ribosomal proteins were accumulated and decreased, respectively. Based on the KEGG (Kyoto Encyclopedia of Genes and Genomes) annotations, these proteins represent 29 ribosomal subunits, but not all of these are major paralogs; the most prominent paralogs form only 50% of the detected isoforms. This indicates that the total ribosome population is not completely altered, or that the alteration is cell-specific, and that this localized distribution was lost in the total shoot protein extracts used in this study. It is noteworthy that the ribosomal subunits did not show mixed-mode response, and that all INCYDE-responsive paralogs for the given subunit always showed the same response.

### 3.3. Positive Effect of INCYDE Could Coincide with Attenuated Stress Response

The response to an exogenous stimuli has been seen to change over time, and it is, thus, not surprising that an early response may be only transient and could significantly differ to that found after prolonged exposure. In the experiment reported here, only 13 early INCYDE response proteins were found to be differentially abundant after 168 h of INCYDE treatment, and only six showed a similar response, including 12-oxo-phytodienoic acid biosynthetic enzyme (AOC2, AT3G25770, repressed) and microsomal prostaglandin E synthase 2 (AT5G42150, repressed). These two enzymes produce substances that trigger plant defense and detoxification response. It has been hypothesized that plants reduce their growth as a primary adaptation response to stress, regardless of the severity of the threat [46], and it is tempting to speculate that the reported INCYDE-promoted growth under suboptimal conditions [23,24,25,26] could coincide with the attenuated stress response. This theory could be supported by an INCYDE-induced attenuation of ABA signaling and repression of at least 21 additional stress-responsive proteins, including UDP-glycosyltransferase 79B2 (AT4G27560, response to cold, drought and salinity; [47]), protein disulfide-isomerase (AT2G32920, response to endoplasmic reticulum stress; [48]) or MD-2-related lipid-recognition protein 3 (AT5G23820, biotic stress, [49]). The comparison of INCYDE response proteins with the database of previously identified phytohormone-responsive proteins [50] found the highest overlap for cytokinin-responsive proteins (representing 18 and 79 proteins for 24 and 168 h treatments, respectively), but the proteins responsive to stress-associated phytohormones ABA and jasmonic acid represented the second and the third most numerous categories, with 82 ABA-responsive proteins and 55 jasmonic acid-responsive proteins, respectively. The INCYDE response was opposite to that of jasmonic acid or ABA treatment for more than 50% of these proteins, supporting the theory that the INCYDE-repressed degradation of cytokinin inhibits stress perception in plants. Further, this fact indicates a higher level of cytokinin interaction with ABA and jasmonic acid than previously reported.

## 4. Materials and Methods 

### 4.1. Plant Material

Seeds of *Arabidopsis thaliana* ecotype Col-0 and transgenic promoter line ARR5::GUS were used for all experiments. Seeds were surface-sterilized and sown on Petri dishes containing half-strength Murashige and Skoog medium with 1% (*w/v*) agar with the corresponding concentration of INCYDE or tZ (10 nM, 100 nM, 1 µM, 10 µM) or 0.1% dimethylsulfoxide (DMSO, mock). Seeds were stratified at 4 °C for three days, and cultivated at 21 °C/19 °C day/night temperatures, with a 16 h photoperiod (100 μmol m^–2^ s^–1^ photosynthetic photon flux density) for up to 14 days in a growth chamber (AR36LX, Percival). For proteomics experiments, seeds were sown on Uhelon 120T (Silk & Progress, Brněnec, Czech Republic) and cultivated as described for mock. After seven days of cultivation, the Uhelon mesh with the seedlings was transferred onto fresh liquid Murashige and Skoog medium supplemented with 0.1% (*v*/*v*) DMSO (mock), tZ or INCYDE in DMSO (final concentration, as for the mock) for five minutes and then transferred onto solidified Murashige and Skoog medium supplemented with the same substance. Plant tissues were harvested after 24 h (whole seedling) and 168 h (shoot tissue) for the analysis of early and prolonged INCYDE response proteins, respectively. The length of the primary root of seven-day-old seedlings was measured using the analysis software ImageJ.

### 4.2. Histochemical Analysis

ARR5::GUS transgenic plants were vacuum infiltrated for 10 minutes with the staining buffer [0.5 M sodium phosphate, 1% (*v/v*) Triton X-114, 0.5 mM potassium ferricyanide, 2 mM potassium ferrocyanide, 1 mg mL^−1^ 5-bromo-4-chloro-3-indolyl-β-d-glucuronide, pH 7.0] and incubated at 37 °C for 2 h. Samples were then incubated in 70% (*v/v*) ethanol and then documented with a camera. The image quantification was performed with ImageJ [51], as described previously [52].

### 4.3. Proteome Analysis

Total protein extracts were prepared as previously described, employing a combination of phenol/acetone/TCA extraction [14]. Portions of samples corresponding to 5 µg of peptide were analyzed by nanoflow reverse-phase liquid chromatography-mass spectrometry using a 15 cm C18 Zorbax column (Agilent), a Dionex Ultimate 3000 RSLC nano-UPLC system and the Orbitrap Fusion Lumos Tribrid Mass Spectrometer (Thermo). Peptides were eluted with up to a 120-min, 4% to 40% acetonitrile gradient. Spectra were acquired using the default settings for peptide identification, employing HCD activation, resolution 60,000 (MS) and 15,000 (MS2), and 60 s dynamic exclusion. The measured spectra were recalibrated and searched against Araport 11 protein database, as described previously [14]. Only proteins with at least two unique peptides were considered for the quantitative analysis. The quantitative differences were determined by Minora, employing precursor ion quantification followed by normalization and background-based t-test for peptide- and protein-based quantitation.

### 4.4. Statistical Analyses

The reported statistical tests were generated and implemented using Instant Clue [53], Rapid Miner (www.rapidminer.com; [54]) and Proteome Discoverer. Significant differences refer to *p* < 0.05.

## 5. Conclusions

Cytokinin metabolism and signaling play important roles in abiotic stress tolerance, and the manipulation of these processes by inhibiting cytokinin degradation could be beneficial for sustainable agriculture. This work provided the first insights into the INCYDE-responsive proteins in *Arabidopsis* seedlings, and found differences between cytokinin tZ and INCYDE effect at the molecular level. The results showed that the INCYDE inhibition of CKX is different from that of cytokinin accumulation in response to exogenous treatment, and found tissue-specific differences in cytokinin signaling. The inhibitory effect of INCYDE on early seedling growth and development was not found in 14-day-old plantlets, indicating that the response is not only tissue-specific, but also developmentally regulated. Finally, the presented data provide evidence of INCYDE-induced stress response attenuation and a new framework for further detailed investigations of the molecular mechanisms involved in hormonal signaling and stress mitigation.

## Figures and Tables

**Table 1:** INCYDE-responsive proteins with significant dose-dependent response. Protein abundance correlation with INCYDE concentration; Pearson’s correlation coefficients r and *p*-value.

Accession	Process	Name	r	*p*-Value
AT4G20260	Calmodulin signaling	Plasma membrane-associated cation-binding protein 1	−0.66	1.5 × 10^−3^
AT3G55800	Carbohydrate metabolism	Sedoheptulose-1,7-bisphosphatase	−0.72	3.4 × 10^−4^
AT2G31390	Carbohydrate metabolism	Fructokinase-1	−0.73	2.7 × 10^−4^
AT1G65960	GABA biosynthesis	Glutamate decarboxylase 2	0.66	1.5 × 10^−3^
AT5G26000	Glucosinolate degradation	Myrosinase 1	0.65	1.7 × 10^−3^
AT5G56010	Chaperon, defense response	Heat shock protein 90-3	0.70	5.9 × 10^−4^
AT4G37000	Chlorophyll biosynthesis	Red chlorophyll catabolite reductase	0.65	1.9 × 10^−3^
AT1G63680	Chloroplast biogenesis	UDP-*N*-acetylmuramoyl-l-alanyl-d-glutamate-2,6-diaminopimelate ligase	0.77	8.1 × 10^−5^
AT1G08520	Chloroplast biogenesis	Magnesium-chelatase subunit	0.68	9.6 × 10^−4^
AT2G04030	Chloroplast biogenesis	Heat shock protein 90-5	0.66	1.5 × 10^−3^
AT3G18420	Chloroplast biogenesis	protein SLOW GREEN 1	−0.65	2.1 × 10^−3^
AT1G72150	Membrane-trafficking	Patellin-1	0.80	1.9 × 10^−5^
AT5G21060	Methionine metabolism	Homoserine dehydrogenase	0.70	6.6 × 10^−4^
AT1G20220	Nucleic acid binding	Alba DNA/RNA-binding protein	−0.65	1.7 × 10^−3^
AT5G60360	Nutrition	Thiol protease aleurain	0.70	6.7 × 10^−4^
AT1G53310	Nutrition, anaplerotic reaction	Phosphoenolpyruvate carboxylase 1	0.68	9.6 × 10^−4^
AT5G43830	Other	DUF3700 domain-containing protein	0.67	1.3 × 10^−3^
AT5G14910	Other	Heavy metal transport/detoxification superfamily protein	−0.67	1.3 × 10^−3^
AT5G44130	Other	Fasciclin-like arabinogalactan protein 13	0.79	3.8 × 10^−5^
AT1G48600	Phospholipid metabolism	Phosphomethylethanolamine N-methyltransferase	0.67	1.2 × 10^−3^
AT5G13120	Photosynthesis	Photosynthetic NDH subunit of lumenal location 5	0.67	1.2 × 10^−3^
AT1G34000	Photosynthesis	Light-harvesting complex-like protein OHP2	−0.68	9.9 × 10^−4^
AT4G38630	Protein degradation	26S proteasome non-ATPase regulatory subunit 4	0.73	2.3 × 10^−4^
AT1G07320	Proteosynthesis	50S ribosomal protein L4, chloroplastic	0.72	3.9 × 10^−4^
AT1G09620	Proteosynthesis	Leucine--tRNA ligase	0.66	1.4 × 10^−3^
AT2G40290	Proteosynthesis	Translation initiation factor eIF-2a	0.65	1.9 × 10^−3^
AT4G29060	Proteosynthesis	Polyprotein of EF-Ts	0.67	1.2 × 10^−3^
AT2G19870	RNA metabolism	tRNA/rRNA methyltransferase	0.93	4.4 × 10^−9^
AT3G55460	RNA metabolism	Serine/arginine-rich SC35-like splicing factor	0.80	2.1 × 10^−5^
AT1G80670	RNA metabolism	Protein RAE1 (RNA export factor 1)	−0.65	2.1 × 10^−3^
AT3G57150	RNA metabolism	H/ACA ribonucleoprotein complex subunit 4	0.67	1.3 × 10^−3^
AT4G09000	Signaling	14-3-3-like protein GF14 chi	−0.65	2.1 × 10^−3^
AT3G44750	Transcription	Histone deacetylase HDT1	0.72	3.3 × 10^−4^

## Supplementary Materials

The following are available online at https://www.mdpi.com/2223-7747/9/11/1563/s1, Figure S1: Representative images of 14-day-old ARR5::GUS reporter line cultivated on the medium supplemented with (i) 0.5 µM trans-Zeatin (tZ) or 0.5 µM INCYDE; Figure S2: Differences in primary root elongation for INCYDE-treated plants after 168 h were not significant; Table S1: Supplementary table to Figure 2a; Table S2: Early INCYDE response proteins, Table S3: Dose-dependent proteome response to INCYDE.
