# Supplementary material for: Arabidopsis Response to Inhibitor of Cytokinin Degradation INCYDE: Modulations of Cytokinin Signaling and Plant Proteome
Source: Plants (Basel). 2020 Nov 13;9(11):1563. doi: 10.3390/plants9111563 (PMC7698199; doi:10.3390/plants9111563)
Supplement: Supplementary file 1 [file plants-09-01563-s001.zip › Supplementary Figures.docx]

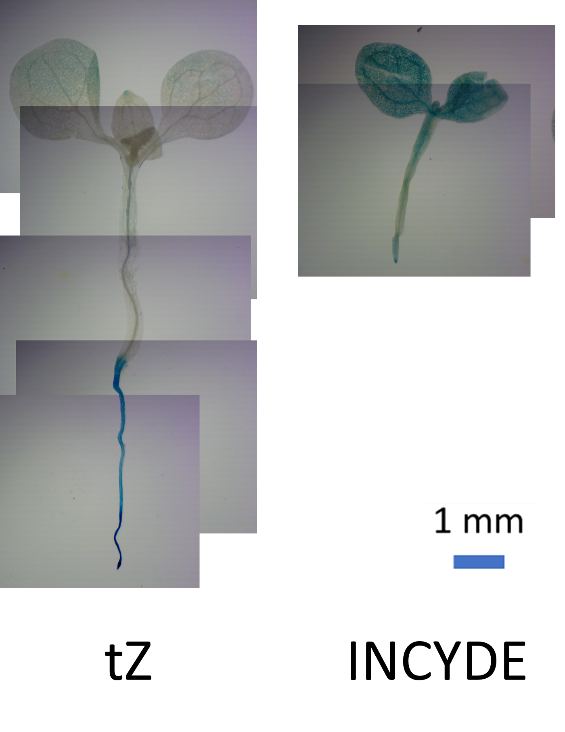


**Figure S1.** Representative images of 14-day-old ARR5::GUS reporter line cultivated on the medium supplemented with (i) 0.5 µM trans-Zeatin (tZ) or 0.5 µM INCYDE.


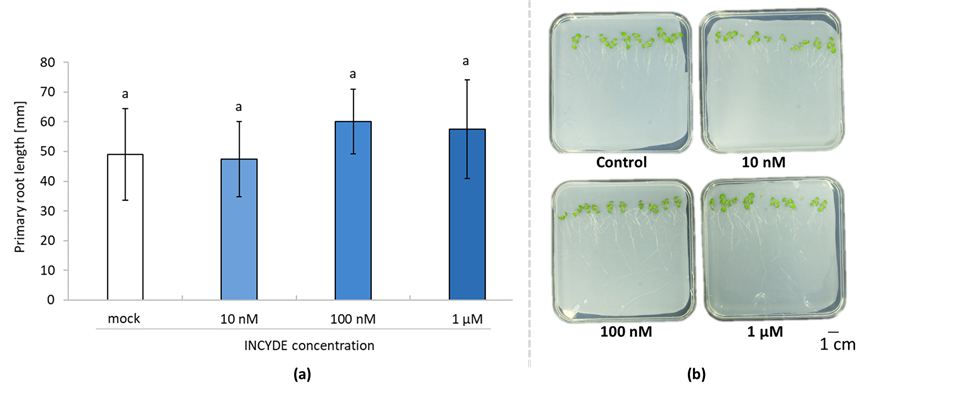


**Figure S2.** Differences in primary root elongation for INCYDE-treated plants after 168h were not significant. (**a**) *Primary root length of 14-day-old plants grown on medium supplemented* with the indicated concentration of INCYDE or DMSO (mock) for seven days; (**b**) Representative images. Results represent means and standard deviation (n = 20), differences are not statistically significant (Kruskal-Wallis, p<0.05).
